# The impact of increased reimbursement rates under the new cooperative medical scheme on the financial burden of tuberculosis patients

**DOI:** 10.1186/s40249-019-0575-z

**Published:** 2019-08-02

**Authors:** Yan-Jiao Xin, Li Xiang, Jun-Nan Jiang, Henry Lucas, Sheng-Lan Tang, Fei Huang

**Affiliations:** 1 School of Medicine and Health Management, Huazhong University of Science and Technology, Wuhan, China; 2 Institute of Development Studies, Brighton, UK; 3 Duke Global Health Institute, Duke University, Durham, NC USA; 4 Global Health Research Center, Duke Kunshan University, Kunshan, China; 5 National Center for Tuberculosis Control and Prevention, China CDC, Beijing, China

**Keywords:** Tuberculosis, New cooperative medical scheme, Financial burden, China

## Abstract

**Background:**

Tuberculosis (TB) is still a major public health problem in China. To scale up TB control, an innovative programme entitled the ‘China-Gates Foundation Collaboration on TB Control in China was initiated in 2009. During the second phase of the project, a policy of increased reimbursement rates under the New Cooperative Medical Scheme (NCMS) was implemented. In this paper, we aim to explore how this reform affects the financial burden on TB patients through comparison with baseline data.

**Methods:**

In two cross-sectional surveys, quantitative data were collected before (January 2010 to December 2012) and after (April 2014 to June 2015) the intervention in the existing NCMS routine data system. Information on all 313 TB inpatients, among which 117 inpatients in the project was collected. Qualitative data collection included 11 focus group discussions. Three main indicators, non-reimbursable expenses rate (NER), effective reimbursement rate (ERR), and out-of-pocket payment (OOP) as a percentage of per capita household income, were used to measure the impact of intervention by comprising post-intervention data with baseline data. The quantitative data were analysed by descriptive analysis and non-parametric tests (Mann-Whitney U test) using SPSS 22.0, and qualitative data were subjected to thematic framework analysis using Nvivo10.

**Results:**

The nominal reimbursement rates for inpatient care were no less than 80% for services within the package. Total inpatient expenses greatly increased, with an average growth rate of 11.3%. For all TB inpatients, the ERR for inpatient care increased from 52 to 66%. Compared with inpatients outside the project, for inpatients covered by the new policy, the ERR was higher (78%), and OOP showed a sharper decline. In addition, their financial burden decreased significantly.

**Conclusions:**

Although the nominal reimbursement rates for inpatient care of TB patients greatly increased under the new reimbursement policy, inpatient OOP expenditure was still a major financial problem for patients. Limited diagnosis and treatment options in county general hospitals and inadequate implementation of the new policy resulted in higher inpatient expenditures and limited reimbursement. Comprehensive control models are needed to effectively decrease the financial burden on all TB patients.

**Electronic supplementary material:**

The online version of this article (10.1186/s40249-019-0575-z) contains supplementary material, which is available to authorized users.

## Multilingual abstracts

Please see Additional file 1 for translations of the abstract into the five official working languages of the United Nations.

## Background

Despite concerted efforts in tuberculosis (TB) control since the 1990s, TB remains a major public health problem in China [1–3]. According to the World Health Organization (WHO) *Global Tuberculosis Report 2018*, China had the second largest estimated incidence in 2017, accounting for 9% of TB cases worldwide. There is a long way to go to meet the goals of WHO’s End TB Strategy and the UN’s Sustainable Development Goals (SDGs) [4]. These are far less than 55 TB cases per 100 000 population and a 75% reduction in TB deaths (compared with 2015) by 2025 and less than 10 TB cases per 100 000 population and a 95% reduction in TB deaths (compared with 2015) by 2035 [5]. Previous studies showed that one of the most common barriers to successful treatment is financial and that this can greatly hinder progress in scaling up care [6–8]. For example, a before-and-after study on the effects of a comprehensive programme for multi-drug resistant tuberculosis (MDRTB) in China showed that 15% of patients had to forgo treatment because of financial difficulties [7]. TB is not only an infectious disease but also a socio-economic problem [6]. Thus, greater financial risk protection measures as well as treatment interventions for patients are needed to achieve universal access to TB care [6].

TB is a poverty-related infectious disease, and a high cost of care may exclude poor patients from the health system and reduce the probability of adequate treatment and cure [9–13]. This may further aggravate the spread of tuberculosis and hinder progress in TB control. Thus, protecting TB patients from financial risk is a priority for policy makers [1]. Over past decades, the academic and policy communities in many countries have proposed and implemented various financial risk protection interventions and pro-poor strategies for TB control programmes to address the needs of the poor, especially in countries with a high TB burden [14]. However, the effectiveness of these programmes has often been limited.

For example, in India, after the implementation of the Revised National Tuberculosis Control Programme (RNTCP), TB remains a major cause of mortality and economic hardship [11]. A study on rural residents in Burkina Faso showed that, despite free tuberculosis care, 75% of interviewed patients faced catastrophic health expenditures [11]. In Rwanda, although the Global Fund against Acquired Immune Deficiency Syndrome, Tuberculosis and Malaria (GFATM)-funded projects greatly improved financial access to health care, most Rwandans still had problems affording the co-payment charged, thus hindering access to TB health services [15].

Globally, there is still a substantial economic burden on many TB patients, which does not conform to the definition of Universal Health Coverage (UHC): that all people receive the health services they need without being exposed to financial hardship [16]. It also runs counter to the goal of the End TB Strategy: that no affected family faces catastrophic costs due to tuberculosis [5].

Although essential TB care is free of charge in China, studies have identified that patients still have to pay significant costs for additional medicine and tests [17–19]. Moreover, under the fee-for-services (FFS) payment typically adopted in China, hospitals are mostly financed by service fees, which leads to perverse incentives for hospitals to offer more services, contributing to higher inpatient expenses and a rapid increase in healthcare costs [20, 21]. Many previous studies have shown that health insurance is a useful tool to protect people from catastrophic health expenditure (CHE) and improve TB treatment completion rates [22–24]. For example, in the United States, TB control was enhanced by bringing uninsured Americans into the health care system [25]. At least one previous study has indicated that integrating the national TB control programme into health insurance schemes was an effective strategy to address current challenges in China [26].

Hence, in the context of the new round of Chinese health system reforms towards UHC, an innovative programme entitled the ‘China-Gates Foundation Collaboration on TB Control in China’ (China-Gates TB Project) was initiated in 2009 by the Chinese National Health and Family Planning Commission and Chinese Center for Disease Control and Prevention (China CDC), with support from the Bill and Melinda Gates Foundation. During the first phase (from 2009 to 2012), the financing model for TB services was changed from a special earmarked fund from the Ministry of Finance to funding mainly provided by health insurance schemes [17]. This proved challenging for the effective financing and delivery of TB services [17]. Thus, two major reforms have been implemented since 2014 during the second phase of the project: increasing the reimbursement rate to 70–80% for TB-related inpatient services and using the case-based payment method to reimburse TB designated hospitals to motivate hospitals to provide standardized treatments and contain costs [17]. However, the extent of any alleviation of the financial burden on TB patients during the implementation of these reforms is unknown. In this paper, we aim to evaluate (i) whether the effective reimbursement rate (ERR) was increased after the improvement in the nominal rate under the NCMS and (ii) the impacts of the new financing model on the financial burden of TB patients covered by the NCMS through comparison with the baseline data.

## Methods

### Study setting

The data used for this paper were derived from evaluation studies for Phase II of the China-Gates Project. This aimed to implement a comprehensive TB/MDRTB control model that could be scaled up over time by the National TB Prevention and Control Program. The survey was conducted in the three cities where the project was implemented (Zhenjiang City, Jiangsu Province; Yichang City, Hubei Province; and Hanzhong City, Shaanxi Province), which are located in the eastern, central and western regions of China, respectively. Three counties (one from each category of high, middle and low GDP per capita) were then selected as study sites in each city. Two preconditions, required by the National Project Office, for TB patients to be included in the project were (1) having health insurance and (2) receiving treatment at a designated hospital. TB patients who did not meet the two preconditions were excluded from the project. This paper chose three counties, representing different levels of socio-economic development, in the central region as our study setting: Zhijiang (ZJ), Yidu (YD), and Wufeng (WF), all within the administrative area of Yichang City.

### Data collection

#### Quantitative data collection

Data were collected by a combination of quantitative and qualitative methods. The focus was on quantitative data with some additional material provided from focus group discussions (FGDs) and document review. Quantitative data were derived from the NCMS. To understand the health expenditures and reimbursements of TB patients, we collected data before (January 2010 to December 2012) and after (April 2014 to June 2015) the intervention from the existing NCMS routine data system in each of the three counties. Eligible patients were identified via a TB diagnosis code. Hospitalization and reimbursement information on 313 TB inpatients were extracted from the NCMS inpatient database. The same information on 117 TB inpatients included in the project, according to the two preconditions mentioned in study setting section, was also extracted from the NCMS inpatient database. To better reflect the effect of the project, the analysis of patients excluded from the project was also added in this paper for comparison with the TB inpatients inside the project. The reimbursement data included the patient’s name, sex, age group, choice of health providers, hospitalization costs and reimbursement.

#### Qualitative data collection

Qualitative information was used to gain a deeper and more complete understanding of the situation. Data collection included FGDs with key stakeholders, including health care providers from the designated hospitals and TB patients. The topic outlines for the FGDs were developed by the principle researchers of the investigation team, and they were conducted by senior academics with rich qualitative research experience. As shown in Table 1, approximately six participants were included in each of 11 FGDs, conducted with the help of local Centers for Disease Control and Prevention (CDC) officials. All were recorded with the permission of the participants. Policy documents relating to the reimbursement of TB patients were also collected from the study sites for review and analysis (Table 1).

**Table 1:** Focus group discussions in Yichang City

FGDs	Participants	Main content
Health care providers	Senior administrators from TB or infectious disease section, health insurance section, and hospital information section; one doctor and one nurse responsible for TB care.	Issues included the financing and expenditure of TB care, provision of TB services, views and suggestions on patient diagnosis and treatment management process and inter-departmental cooperation mechanism
TB patients	Six patients who had completed a treatment course and could clearly express their thoughts. Sex, age group, geographic location of residence, and socio-economic status were considered.	Questions regarding their diagnosis and treatment, expenses and reimbursement related to TB care, and their subjective feelings about their financial burden and the impacts of the project on their financial burden.

*FGDs* Focus group discussions, *TB* Tuberculosis

### Data analysis

#### Quantitative data analysis

The quantitative data were analysed using SPSS (version 22.0 Statistical software, International Business Machines Corporation, New York City, US). The main analysis focused on the expenditure and reimbursement for TB-related services and the change in effective reimbursement rates between the baseline and the intervention period. In this paper, we defined the actual implementation rate of the new policy as the number of TB inpatients reimbursed by the new policy as a percentage of total TB inpatients. Total medical expenditure was defined as the sum of reimbursement and any OOP payments. OOP payments were composed of the expenses co-paid by patients and the non-reimbursable amount that exceeded the NCMS benefit packages. The effective reimbursement rate (ERR) was defined as the actual reimbursement amount divided by the total medical expenditure [27]. The non-reimbursable expenses rate (NER) was defined as the non-reimbursable amount beyond the NCMS benefit packages divided by the total medical expenditure. The nominal reimbursement rate was stipulated in the project under the NCMS. We used the ratio of OOP to the per capita income of rural residents to measure the financial burden of TB patients.

A descriptive analysis of the average medical expenses, OOP, NER, ERR and OOP/per capita income of rural residents was performed. To assess the impact of the project on the financial burden of TB patients, non-parametric tests (Mann-Whitney U test) were employed to identify differences in effective reimbursement rates and the ratio of OOP to per capita income of rural residents in the baseline and intervention periodperiods. We compared the financial burden of all TB patients and TB patients included in the project in the intervention to those in the baseline in three counties. *P* < 0.05 was considered statistically significant.

#### Qualitative data analysis

The qualitative data from the FGDs were subjected to thematic framework analysis using.

NVivo (version 10, 0 QSR International, Melbourne, Australia). First, the theme of each level initially according to the interview outline. Then, developing, a topic-stakeholders table was developed, and encoded the contents of the text information were encoded into the corresponding cells of the table to present the diverse views of different stakeholders on the same topic. In addition, new themes were identified, and the framework was further improved. After the encoding process, the contents of the two-dimensional table were summarized, and themes were formulated. Finally, the formulated themes were checked against the coded extracts to ensure that they captured the key issues raised by the participants.

As shown in Table 2, four themes were formulated: the TB prevention and control system, the implementation of the increased reimbursement, the impact of the new policy, and regulation of that policy. These four themes were checked against the coded extracts to ensure that they captured the key issues raised by the participants.

**Table 2:** Thematic framework analysis using FGD data

First level	Second level
TB prevention and control system	a. Functional transformation of relevant departments under the project b. Health services provided by designated hospitals in the project c. The responsibilities of the Chinese Center for Disease Control and Prevention d. The responsibilities of village doctors
The implementation of the new policy	a. Financing policy b. Payment policy c. Publicity and patients’ awareness of the new policy
The impact of the new policy	a. Impacts on total cost control for TB treatment b. Impacts on patients’ financial burden c. Views on the new financing and payment policy
Regulation of the implementation of the new policy	a. Regulation of designated hospitals b. Regulation of medical staff behaviour in hospitals c. Regulation of village doctors

*FGD* Focus group discussion, *TB* Tuberculosis

## Results

### Reimbursement policy for TB patients under the NCMS in Yichang City

Using health insurance policy documents and the FGDs, we analysed the inpatient service reimbursement policy for TB patients under the NCMS at baseline and during the intervention period. At baseline, the NCMS was decentralized and administered at the county level [28]. Since TB patients must now seek treatment in higher-level hospitals, the old reimbursement policy restricted the effective reimbursement rate.

In the intervention period, the reimbursement policy for TB patients was unified at the municipal level. As shown in Table 3, nominal reimbursement rates for smear-negative inpatients and smear-positive inpatients were 80 and 90% within the package, respectively. For the actual implementation rate of the new policy Yidu and Wufeng had relatively more effective implementation, with actual reimbursement rates higher than 70% (Table 3).

**Table 3:** New reimbursement policy for TB inpatients under the NCMS in Yichang City

Nominal reimbursement rate		Actual implementation rate of new reimbursement policy
Baseline	Intervention	Intervention
Township health centers: ZJ: 85%, YD: 85%, WF: 80%. County hospitals: ZJ: 75%, YD:65%, WF:70%. Municipal hospital: ZJ: 50%–65%, YD: 50–65%, WF: 55-65%.	Smear-negative patients: 80%; Smear-positive patients: 90%	ZJ: 55%; YD: 70%; WF: 74%

Data sources: collected from policy documents from health bureaus and health insurance agencies

*TB* Tuberculosis, *NCMS* New Cooperative Medical Scheme, *ZJ* Zhijiang City, *YD* Yidu city, *WF* Wufeng City

### TB inpatient care reimbursement under the NCMS in Yichang City

Reimbursement for TB inpatients under the NCMS before and after intervention.

As shown in Table 4, according to the NCMS database, 313 TB inpatients were reimbursed in the post-intervention period in Yichang City compared with 1001 at baseline. For all TB patients, the mean inpatient expenses had greatly increased, from RMB 5067 to RMB 5 638, an average growth rate of 11.3%. The effective reimbursement rate over the intervention period was 66%, significantly higher than that at the baseline (52%) (Mann-Whitney U test *z* = − 10.854, *P* < 0.001), while OOP and NER showed a gentle decrease, but to different degrees across the three counties. The expenses co-paid by patients decreased greatly, while non-reimbursable expenses had a slight increase.

**Table 4:** Reimbursement for TB inpatients under the NCMS before and after intervention

Residence	Term	Number	Mean inpatient expenses (RMB)	OOP (RMB)			NER (%)	ERR (%)
				Total	Co-paid	Non-reimbursable		
ZJ	Baseline	242	6372	3048	2220	828	13	52
	Intervention	76	6231	2034	1286	748	12	67^***^
YD	Baseline	430	4522	2397	2035	362	8	47
	Intervention	150	5508	2135	1639	496	9	61^***^
WF	Baseline	329	4818	2076	1787	289	6	57
	Intervention	87	5344	1495	1281	214	4	72^***^
Total	Baseline	1001	5067	2450	1994	456	9	52
	Intervention	313	5638	1933	1454	479	8	66^***^

Data source: NCMS database

*TB* Tuberculosis, *NCMS* New Cooperative Medical Scheme, *ZJ* Zhijiang City, *YD* Yidu City, *WF* Wufeng City, *OOP* Out-of-pocket payment, *NER* Non-reimbursable expenses rate, *ERR* Effective reimbursement rate

^***^: significant at the 1% levels

Reimbursement for TB inpatients inside and outside the project after intervention.

As shown in Tables 5, 117 TB inpatients were included in the project, and 196 TB inpatients were excluded from the project in the post-intervention period. Compared with TB patients outside the project, patients inside the project had higher inpatient expenses and ERR (Mann-Whitney U test *z* = − 13.455, *P* < 0.001).

**Table 5:** Reimbursement for TB inpatients inside and outside the project after intervention

Residence	Patients	Number	Mean inpatient expenses (RMB)	OOP (RMB)	ERR (%)
ZJ	Intervention-a	41	4859	917	81
	Intervention-b	35	7838	3342	57^***^
YD	Intervention-a	48	6602	1748	74
	Intervention-b	102	4993	2317	53^***^
WF	Intervention-a	28	5740	1064	81
	Intervention-b	59	5156	1700	67^***^
Total	Intervention-a	117	5785	1293	78
	Intervention-b	196	5550	2315	59^***^

Data source: NCMS database

Note: intervention-a indicates reimbursement data for TB patients included in the project and intervention-b reimbursement data for TB patients excluded from the project

*TB* Tuberculosis, *ZJ* Zhijiang City, YD Yidu City, *WF* Wufeng City, *OOP* Out-of-pocket payment, *ERR* Effective reimbursement rate

^***^: significant at the 1% levels

**Fig. 1:**
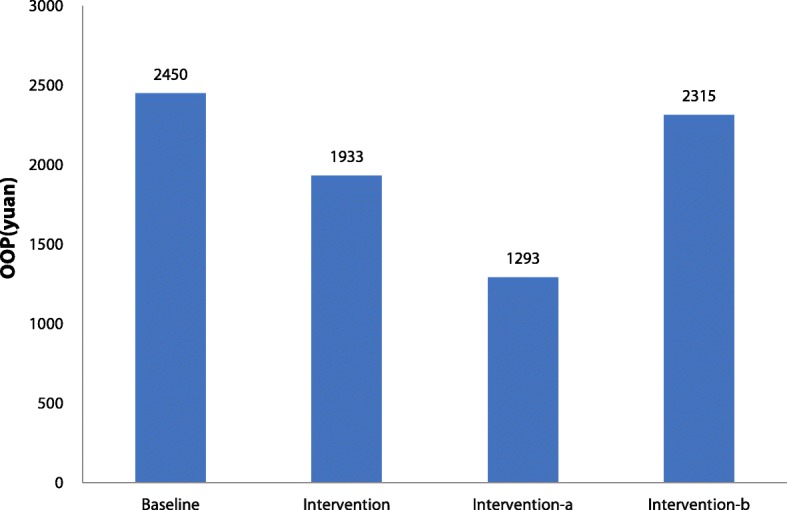
Mean out-of-pocket payment for Tuberculosis inpatients before and after intervention Note: interview-a indicates reimbursement data for TB patients included in the project; Intervention-b reimbursement data for TB patients excluded from the project; OOP: out-of-pocket payment

**Fig. 2:**
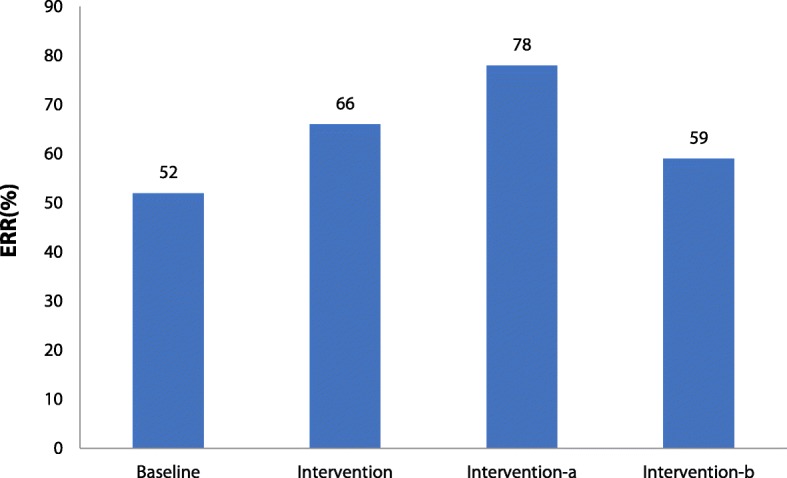
The effective reimbursement rate for Tuberculosos inpatients before and after intervention Note: intervention-a indicates reimbursement data for TB patients included in the project; Intervention-b reimbursement data for TB patients excluded from the project; ERR: effective reimbursement rate

Figures 1 and 2 show the mean OOP and ERR for TB inpatient care in the baseline and intervention periods. Across all TB patients, the mean OOP decreased greatly compared with baseline. However, the mean OOP for TB patients outside the project showed a gentle decrease, while that for TB patients in the project showed a sharp decline. Similarly, for TB patients included in the project, the ERR was significantly higher than for patients outside the project.

### Impact of the NCMS on TB financial burden

The financial burden of TB patients was expressed in terms of OOP as a percentage of per capita household income. As shown in Table 6, inpatient care expenditure remained a major financial burden for TB patients. For all TB inpatients, the financial burden decreased in all three counties but differed greatly, with Wufeng having the heaviest burden (15%). The financial burden for patients in the project significantly decreased (Mann-Whitney U test *z* = − 11.297, *P* < 0.001) and was substantially lower than that for patients excludedfrom the project.

**Table 6:** Financial burden of TB patients before and after intervention

residence	Mean per capita income of rural residents (RMB)		OOP/per capita income of rural resident (%)			
	baseline	intervention	baseline	intervention	intervention-a	intervention-b
ZJ	9169	15 285	33	13^***^	6^***^	22^***^
YD	9096	15 035	26	14^**^	12^***^	15
WF	3815	7146	54	21^***^	15^***^	24
Total	7027	11 837	35	16^***^	11^***^	20^**^

Data source: NCMS database

Note: intervention-a indicates reimbursement data for TB patients included in the project and intervention-b reimbursement data for TB patients excluded from the project

*TB* Tuberculosis, *ZJ* Zhijiang City, *YD* Yidu City, *WF* Wufeng City, *OOP* Out-of-pocket payment

^**^, ^***^: significant at the 5, and 1% levels, respectively

## Discussion

In this study, we found that the nominal reimbursement rates for inpatient care of TB patients greatly increased to 80–90%, as shown in Table 3, under the new reimbursement policy in the project. However, for inpatient care, although the ERR for TB patients included in the project increased significantly, the impact of the new policy on all TB inpatients and TB inpatients outside the project was limited. Inpatient OOP expenditure for TB care remains a major financial problem for TB patients.

Although the new policy greatly improved the ERR, there was still a gap between the nominal and effective reimbursement rates. For inpatient care, the ERR for patients included in the project was significantly higher than post-intervention, and after reimbursement from the NCMS, OOP payments significantly decreased. However, for all TB patients the ERR only marginally increased, OOP payments were considerably higher in the intervention period. The reduced ERRs remain financial barriers to health services, as TB is a poverty-related disease [29].

The limited effect of the intervention on the totality of TB patients could be attributed to a number of factors. First, the fact that some TB patients were excluded from the project limited the effect of our project. From our results, we found that patients outside the project had higher OOP and lower ERR and that the financial burden for TB inpatients outside the project was substantially heavier than that for TB inpatients in the project post-intervention. Analysis of policy documents indicated that the two preconditions specified above excluded somesome uninsured TB patients and patients receiving treatment at non-designated hospitals from the project. Additionally, the FGDs showed that in Yichang City, some unreasonable human factors limited the range of intervention. For example, in YD, patients with complications were typically admitted under other diagnoses and were thus also excluded. Some designated hospitals excluded patients with high expenditures exceeding the quota of the case-based payment from the project. Second, comprehensive policy implementation was required. Although the three selected counties were in principle implementing the same reimbursement policy, the resulting ERRs varied considerably. Analysis suggests that low levels of reimbursement correlated with limited policy implementation. Taking Zhijiang as an example, its policy implementation rate, which was only 55%, was the lowest among the three counties, and its effective reimbursement rate was only 67%.

The fact that more inpatients sought medical care at municipal or higher-level hospitals may be another factor lowering the overall ERR. This not only results in higher inpatient expenditures by TB patients, for example, on supplementary drugs and additional tests, but can also restrict reimbursement for inpatient care because it tends to encourage a more cautious and restrictive approach by the NCMS, meaning that TB patients hospitalized at the municipal or higher-level hospitals may often have been excluded from the intervention. To some extent, the increase in the use of higher-level hospitals is probably related to a perception among patients that county general hospitals have more limited diagnosis and treatment options and less highly qualified staff.

With major improvements in infrastructure, it has become much easier for TB patients to travel to municipal or higher-level hospitals. The ever increasing use of higher-level facilities may well continue if there is no measure to control access. Comprehensive TB control models for the effective diagnosis, treatment and management of TB patients will require further efforts to enhance professional and health education programmes at the county level [17, 21, 30]. It may also be the case that county-level designated hospitals are refusing to treat patients with relatively severe illness whose treatment cost might exceed the standard for a single disease under the case-based payment mechanism. This question requires further research.

Although the ERR for TB inpatient care for those included in the project improved significantly and their OOP payment decreased substantially, OOP as a percentage of per capita household income for rural residents still often exceeded 10%, usually taken as the threshold of catastrophic health expenditure [1]. For TB inpatients excluded from the project, the situation was considerably worse. The financial burden between TB inpatients inside and outside the project was remarkably different: TB inpatients outside the project had a heavier financial burden than TB inpatients in the project. This is partly a reflection of the fact that TB is a poverty-related disease, with most TB patients having low socioeconomic status. The total health expenditure post-intervention increased remarkably. One reason may be that improvement in the reimbursement rate for TB patients promoted an increase in the demand for TB services and treatment compliance [31]. Some studies have shown that poverty is a main factor restricting the utilization of TB health services, and failure to adhere to the complete treatment cycle of TB care still exists in many areas of China [19, 32]. Note that, as TB disproportionally affects the poor population in less-developed regions, patients may suffer an additional financial burden due to interrupted and/or inappropriate treatment [33], which will also impact non-medical costs, including travel, subsistence and loss of earnings.

Cost control is essential to reduce the cost of care and the risk of a range of serious problems, including treatment delays, poor treatment adherence and drug resistance [1, 19, 34]. It seems clear that poor implementation of the new policy weakened the intended impact on cost control. Case-based payments were the preferred option. Compared with a fee-for-services approach, this was seen as one way to reduce the provision of unnecessary services [9, 31]. However, inadequate implementation weakened any impact of cost control, contributing to the high level of OOP.

This result may have been influenced by the attitudes of some key actors. Several CDC directors suggested that it was unfair to patients with other diseases to increase reimbursement for TB alone. Limited health service packages that excluded some essential diagnosis and treatment services were another reported reason for ineffective implementation of the new policy. Officials of health insurance agencies indicated that they thought that the package reflected a high standard of treatment and emphasized that they had not been involved in its formulation. They simply implemented the policy and suggested that the design might have been improved if they had participated. However, designated hospital managers expressed the opinion that using the average cost of TB treatment over the previous 3 years as the basis for the standard package was too simplistic. The cost for a considerable number of patients with severe illness or complications substantially exceeded this estimate. Some participants argued that the revenue targets set for their hospital could not be achieved under the new policy and that the limited health service packages might constrain clinical options and cause great concern to doctors who felt they were not providing the optimal treatment. Given the negative attitudes of some health service providers, a generally agreed standard, improved monitoring and effective supervision are needed to guarantee that the policy reforms are implemented as intended.

### Limitations

First, we only evaluated the quantitative implications of the project from the perspective of patients. The impact on service providers (doctors, designated hospitals, government officials, etc.) are equally important but were not analysed in the context of the new policy. Second, as we discussed, some unreasonable human factors excluded TB inpatients from the project, which may influence the conclusion. However, the percentages of patients excluded from project with different reason were unobtainable from the data we have. And our further research will focus on this point.

## Conclusions

Although the nominal reimbursement rates for inpatient care of TB patients greatly increased following the intervention, there was still a gap between the nominal and effective reimbursement rates. The exclusion of many patients from the benefits of the intervention considerably reduced its impact. Inpatient OOP expenditure remained a major financial problem for patients, partly because TB is a poverty-related disease. Comprehensive TB control models, including enhancing professional and health education programmes at the county level, setting generally agreed standards, improving monitoring and ensuring effective supervision, are needed to guarantee that such projects are implemented as intended and effectively decrease the financial burden of all TB patients.

## Additional file

Additional file 1: Multilingual abstracts in the five official working languages of the United Nations. (PDF 217 kb)

## Data Availability

The datasets used and analysed during the current study are available from the corresponding authors upon reasonable request.

## Abbreviations

CHE: Catastrophic health expenditure
ERR: Effective reimbursement rate
FFS: Fee for services
FGDs: Focus group discussions
MDRTB: Multi-drug resistant tuberculosis
NCMS: New Cooperative Medical Scheme
NER: Non-reimbursable expenses rate
OOP: Out-of-pocket payment
TB: Tuberculosis
UHC: Universal Health Coverage
WF: Wufeng County
YD: Yidu County
ZJ: Zhijiang County
