# Supplementary material for: The impact of increased reimbursement rates under the new cooperative medical scheme on the financial burden of tuberculosis patients
Source: Infect Dis Poverty. 2019 Aug 2;8:67. doi: 10.1186/s40249-019-0575-z (PMC6676612; doi:10.1186/s40249-019-0575-z)
Supplement: Supplementary file 1 — Multilingual abstracts in the five official working languages of the United Nations. (PDF 217 kb) [file 40249_2019_575_MOESM1_ESM.pdf]

Translation of the abstract into the five official working languages of the United Nations

تأثير زيادة معدلات السداد بموجب الخطة الطبية التعاونية الجديدة على العبء المالي بسبب مرضى السل

يان-بياو شين ولي شيانج وجون-نان جيانج وهنري لوكاس وشنج-لان تانج وفاي هوانج

الملخص
معلومات أساسية: لا يزال السل (TB) يمثل مشكلة صحية عامة رئيسية في الصين. ففي عام 2009، لزيادة مكافحة السل، أُطلِق برنامج مبتكر بعنوان "تعاون مؤسسة بوابات-الصين لمكافحة السل في الصين". خلال المرحلة الثانية من المشروع، تم تنفيذ سياسة لزيادة معدلات السداد بموجب الخطة الطبية التعاونية الجديدة (NCMS). في هذا البحث، نهدف إلى استكشاف كيفية تأثير هذا الإصلاح على العبء المالي على مرضى السل من خلال المقارنة مع البيانات الأساسية.
الوسائل:في مسحين مستعرضين، جُمعت البيانات الكمية قبل (من يناير 2010 إلى ديسمبر 2012) وبعد (من أبريل 2014 إلى يونيو 2015) التدخل في نظام البيانات الروتينية الحالية NCMS. جُمعت معلومات عن جميع مرضى السل 313، ومن بينهم 117 مريضًا في المشروع. وشملت عملية جمع البيانات النوعية مناقشة لمناقشات 11 مجموعة التركيز. أُستُخدِمت ثلاثة مؤشرات رئيسية، وهي معدل المصروفات غير القابلة للاسترداد (NER)، ومعدل السداد الفعلي (ERR)، والدفع من الجيب (OOP) كونهم نسبة مئوية من دخل الأسرة الفردي، لقياس تأثير التدخل من خلال تضمين بيانات ما بعد التدخل مع البيانات الأساسية. حُلِلت البيانات الكمية باستخدام SPSS 22.0، وخُضعت البيانات النوعية لتحليل الإطار المواضيعي باستخدام Nvivo10.
النتائج: كانت معدلات السداد للأسماء المرشحة للرعاية الداخلية للمرضى لا تقل عن 80٪ للخدمات داخل الحزمة. وزاد إجمالي نفقات إقامة المرضى زيادة كبيرة، حيث بلغ متوسط معدل الزيادة نحو 11.3٪. بالنسبة لجميع مرضى السل المقيمين، زاد معدل السداد الفعلي في الرعاية الداخلية للمرضى من 52٪ إلى 66٪. مقارنةً بالمرضى المقيمين خارج المشروع، كان معدل السداد الفعلي للمرضى المقيمين المشمولين بالسياسة الجديدة أعلى (78٪)، وأظهر الدفع من الجيب انخفاضًا حادًا. بالإضافة إلى ذلك، انخفض العبء المالي عليهم بشكل كبير.
الاستنتاجات: فعلى الرغم من أن معدلات السداد للأسماء المرشحة للرعاية الداخلية لمرضى السل زادت بشكل كبير في ظل سياسة السداد الجديدة، فإن نفقات الدفع من الجيب للمرضى المقيمين لا تزال تمثل مشكلة مالية كبيرة للمرضى. وأدت خيارات التشخيص والعلاج المحدودة في المستشفيات العامة بالمقاطعة وعدم التنفيذ الكافي للسياسة الجديدة إلى ارتفاع نفقات المرضى المقيمين ومحدودية السداد. هناك حاجة إلى نماذج تحكم شاملة لتقليل العبء المالي على جميع مرضى السل بشكل فعال.

Translated from English version into Arabic by Amal Imam, Revised by Fatma Ossama, through

TRANSLATORS
WITHOUT BORDERS

提高新型农村合作医疗制度报销比例对肺结核患者经济负担的影响分析

摘要

引言：结核病目前仍然是中国公共卫生领域的重大问题。为进一步加强结核病控制，2009 年在中国农村试点地区启动了“中国-盖茨基金会结核病防治合作”项目。项目实施的第二阶段，提高了新农合的报销比例，本文旨在通过与基线数据的比较，探讨这一干预对结核病患者经济负担的影响。

方法：通过两次横截面调查，分别收集了干预前后湖北省宜昌市三个县新农合报销数据（2010.01-2012.12 和 2014.04-2015.06）。共收集了干预后 313 名肺结核住院患者的信息，其

中项目内有 117 名住院患者。采用不可报销费用比例(NER)、实际补偿比(ERR)和个人自付费用(OOP)三个主要指标，通过和基线数据比较，衡量项目实施效果。定性资料收集包括 11 个焦点小组讨论。定量数据采用 SPSS 22.0 进行分析，定性数据采用 Nvivo10 进行主题框架分析。
结果：结核患者报销目录内的医疗费用政策报销比例不低于 80%。与基线相比，三个地区总医疗费用平均上升了 11.3%。肺结核住院患者的实际补偿比从 52%提高到 66%。与项目外患者相比，项目内肺结核患者的实际补偿比更高（78%），个人自付费用明显下降，疾病经济负担显著减轻。
结论：项目虽然提高了肺结核患者住院费用的政策报销比例，但住院患者个人自付费用负担仍然较重。县级医疗机构有限的诊疗水平和项目的未充分落实，导致了较高的医疗费用和有限的报销水平。需要采取综合性的干预措施，来有效降低肺结核患者的疾病经济负担。

Translated from English version into Chinese by Fei Huang

L'impact de l'augmentation des taux de remboursement en vertu du nouveau système de santé coopératif sur la charge financière des patients tuberculeux

Yan-Yiao Xin, Li Xiang, Jun-Nan Jiang, Henry Lucas, Sheng-Lan Tang et Fei Huang

Résumé
Contexte: La tuberculose (TB) est encore un problème majeur de santé publique en Chine. Afin de renforcer la lutte antituberculeuse, un programme novateur intitulé « Collaboration de la Fondation Chine-Gates sur la lutte antituberculeuse en Chine » a été lancé en 2009. Au cours de la deuxième phase du projet, une politique d'augmentation des taux de remboursement en vertu du nouveau système de santé coopératif (MR) a été mis en œuvre. Dans ce document, nous avons pour objectif d'étudier comment cette réforme a une incidence sur la charge financière des patients atteints de tuberculose par rapport aux données de référence.
Méthodes : Dans deux enquêtes transversales, les données quantitatives ont été recueillies avant (janvier 2010 à décembre 2012) et après (avril 2014 à juin 2015) l'intervention de l'actuel système de données de routine de MR. Des informations sur les 313 patients de la tuberculose, dont 117 patients hospitalisés dans le projet ont été recueillies. La collecte des données qualitatives inclus 11 groupes de discussion. Trois indicateurs principaux, des dépenses non-remboursables (TNS), taux de remboursement efficace (ERR), et des paiements directs (POO) en pourcentage du revenu des ménages par habitant, ont été utilisés pour mesurer l'impact de l'intervention comprenant des données post-intervention avec des données de base. Les données quantitatives ont été analysées à l'aide de SPSS 22.0, et des données qualitatives ont été soumis à l'analyse du cadre thématique à l'aide de Nvivo10.
Résultats: La valeur nominale de taux de remboursement des soins hospitaliers n'était pas moins de 80 % pour les services à l'intérieur de l'emballage. Les frais d'hospitalisation Total a fortement augmenté, avec un taux de croissance moyen de 11,3 %. Pour tous les patients de la tuberculose, l'ERR pour des soins en milieu hospitalier est passé de 52 % à 66 %. Par rapport aux patients hospitalisés à l'extérieur du projet, pour les patients hospitalisés couverts par la nouvelle politique, l'ERR était plus élevé (78 %), et de la POO a montré une baisse plus. En outre, leur fardeau financier

a diminué sensiblement.
Conclusions: Bien que le taux de remboursement de valeur nominale pour les soins hospitaliers de patients tuberculeux ait considérablement augmenté dans le cadre de la nouvelle politique de remboursement des dépenses d'hospitalisation, d'OOP était encore un problème financier majeur pour les patients. Le diagnostic et les options de traitement limitées dans les hôpitaux généraux du comté et l'insuffisance de la mise en œuvre de la nouvelle politique ont entraîné une hausse des dépenses d'hospitalisation et remboursement limité. Les modèles de contrôle global est nécessaire pour réduire efficacement la charge financière sur tous les patients atteints de tuberculose.

Translated from English version into French by Emilie Rigault Fourcadier, Revised by Iris Soliman, through

TRANSLATORS
WITHOUT BORDERS

Влияние повышения ставок возмещения расходов в рамках новой кооперационной схемы медицинского страхования при недостатке финансовых средств на лечение пациентов с туберкулезом

Янь-Яо Синь, Ли Сян, Цзюнь-Нань Цзян, Генри Лукас, Шен-Лань Тан и Фэй Хуан

Аннотация

Справочная информация: Туберкулёз (ТБ) по-прежнему представляет собой значительную проблему в области здравоохранения в Китае. Для расширения борьбы с ТБ в 2009 г. была запущена инновационная программа под названием "Сотрудничество Китая и Фонда Гейта по борьбе с туберкулезом в Китае". На втором этапе данного проекта была введена политика повышения ставок возмещения расходов в рамках новой кооперационной схемы медицинского страхования (НКСМС). Цель данного документа — изучить механизмы влияния недостатка финансовых средств на лечение пациентов с ТБ путем сравнительного сопоставления с исходными данными.
Методы: С помощью двух перекрестных исследований были собраны количественные данные до (с января 2010 г. по декабрь 2012 г.) и после (с апреля 2014 г. по июнь 2015 г.) внесения изменений в существующую систему регулярно собираемых данных в рамках НКСМС. Была собрана информация по всем из 313 стационарных пациентов с ТБ, при этом из них в проекте принимало участие 117 стационарных пациентов. Сбор качественных данных охватывал 11 обсуждений в фокус-группах. Три ключевых показателя: предоставление финансовых средств на безвозмездной основе, эффективная ставка возмещения и оплата за счет личных средств в процентах от месячного дохода семьи используются для оценки влияния мероприятий с помощью сопоставления данных, полученных после их проведения, с исходными данными.Анализ количественных данных проводился с помощью программы SPSS 22.0, а предметный рамочный анализ качественных данных — с помощью Nvivo10.

Результаты: Первичные ставки возмещения расходов на лечение стационарных пациентов составили не менее чем 80 % за предоставление услуг в рамках пакета услуг. Общие затраты на лечение стационарных пациентов значительно увеличились, при среднем показателе роста в 11,3 %. Для всех пациентов с ТБ эффективная ставка возмещения расходов на лечение стационарных больных увеличилась с 52 % до 66 %. По сравнению со стационарными больными вне рамок данного проекта, для стационарных пациентов в рамках новой политики эффективная ставка возмещения была выше (78 %) и случаи оплаты за счет личных средств резко уменьшились. Кроме того, финансовые затраты значительно сократились.
Выводы: Несмотря на то, что номинальная ставка возмещения расходов на лечение пациентов с ТБ в стационаре значительно увеличилась под влиянием новой политики возмещения расходов, необходимость оплаты затрат на лечение стационарных больных за счет личных средств по-прежнему является основной финансовой проблемой для пациентов. Ограниченность методов диагностики и вариантов лечения в провинциальных больницах общего профиля, а также недостаточная реализация новой политики привели к повышению затрат на лечение стационарных пациентов и ограничению возмещения расходов. Для эффективного сокращения финансовых затрат на лечение всех пациентов с ТБ необходимо разработать модели комплексной борьбы с заболеванием.

Translated from English version into Russian by Veronika Demeshchyk, Revised by Michael Orlov, through

TRANSLATORS
WITHOUT BORDERS

El impacto sobre la carga financiera de los pacientes de tuberculosis del aumento en las tasas de reembolso bajo el Nuevo Sistema Médico Cooperativo de China

Yan-Yiao Xin, Li Xiang, Jun-Nan Jiang, Henry Lucas, Sheng-Lan Tang y Fei Huang

Resumen
Contexto: La tuberculosis (TB) sigue siendo un importante problema de salud pública en China. En 2009, con el objetivo de ampliar el control de la tuberculosis, se inició un programa innovador llamado Colaboración China - Fundación Gates para el Control de la Tuberculosis en China. Durante la segunda fase del proyecto, se implementó una política de aumento de las tasas de reembolso bajo el Nuevo Sistema Médico Cooperativo (NCMS, por sus siglas en inglés). El objetivo del presente artículo es explorar cómo esta reforma afecta la carga financiera de los pacientes con TB, por medio de la comparación con los datos de referencia.
Métodos: Se recopilaron datos cuantitativos en dos encuestas transversales antes (de enero de 2010 a diciembre de 2012) y después (de abril de 2014 a junio de 2015) de la intervención en el sistema existente de datos rutinarios del NCMS. Se recopiló información sobre los 313 pacientes con tuberculosis hospitalizados; entre ellos, 117 pacientes hospitalizados dentro del proyecto. La recopilación de datos cualitativos incluyó 11 discusiones de grupos de debate. Se utilizaron tres

indicadores principales para medir el impacto de la intervención al integrar los datos de postintervención con los datos de referencia: la tasa de gastos no reembolsables (NER), la tasa de reembolso efectiva (ERR) y los costos de bolsillo propio (OOP) como porcentaje del ingreso per cápita de los hogares. Los datos cuantitativos se analizaron con SPSS 22.0 y los datos cualitativos fueron objeto de un análisis de marco temático mediante el uso de Nvivo10.

Resultados: Las tasas de reembolso nominales para la atención hospitalaria no fueron inferiores a un 80 % para los servicios dentro del paquete. Los gastos intrahospitalarios totales aumentaron considerablemente, con una tasa promedio de crecimiento del 11,3 %. La ERR de la atención hospitalaria aumentó del 52 % al 66 % para todos los pacientes con TB hospitalizados. En comparación con los pacientes hospitalizados no incluidos en el proyecto, la ERR fue mayor (78 %) en los pacientes cubiertos por la nueva política, y los OOP mostraron un descenso más marcado. Además, su carga financiera disminuyó significativamente.

Conclusiones: Aunque las tasas de reembolso nominales para la atención hospitalaria de pacientes con TB aumentaron considerablemente con la nueva política de reembolso, los OOP intrahospitalarios continuaban siendo un problema financiero importante para los pacientes. Las limitadas opciones para el diagnóstico y tratamiento en los hospitales generales del condado y la inadecuada implementación de la nueva política dieron como resultado mayores gastos intrahospitalarios y un reembolso limitado. Se necesitan modelos de control integrales para disminuir de forma efectiva la carga financiera en todos los pacientes con TB.

Translated from English version into Spanish by Christian Bertin, Revised by Maria Paula Gorgone, through

TRANSLATORS
WITHOUT BORDERS
